# Comparison of ground reaction forces as running speed increases between male and female runners

**DOI:** 10.3389/fbioe.2024.1378284

**Published:** 2024-07-29

**Authors:** Xinyan Jiang, István Bíró, József Sárosi, Yufei Fang, Yaodong Gu

**Affiliations:** ^1^ Department of Radiology, Ningbo No. 2 Hospital, Ningbo, China; ^2^ Doctoral School on Safety and Security Sciences, Obuda University, Budapest, Hungary; ^3^ Faculty of Engineering, University of Szeged, Szeged, Hungary; ^4^ Faculty of Sports Science, Ningbo University, Ningbo, China

**Keywords:** gender, running speed, biomechanics, impulse, ground reaction force

## Abstract

**Introduction:** The biomechanics associated with human running are affected by gender and speed. Knowledge regarding ground reaction force (GRF) at various running speeds is pivotal for the prevention of injuries related to running. This study aimed to investigate the gait pattern differences between males and females while running at different speeds, and to verify the relationship between GRFs and running speed among both males and females.

**Methods:** GRF data were collected from forty-eight participants (thirty male runners and eighteen female runners) while running on an overground runway at seven discrete speeds: 10, 11, 12, 13, 14, 15 and 16 km/h.

**Results:** The ANOVA results showed that running speed had a significant effect (*p* < 0.05) on GRFs, propulsive and vertical forces increased with increasing speed. An independent t-test also showed significant differences (*p* < 0.05) in vertical and anterior-posterior GRFs at all running speeds, specifically, female runners demonstrated higher propulsive and vertical forces than males during the late stance phase of running. Pearson correlation and stepwise multiple linear regression showed significant correlations between running speed and the GRF variables.

**Discussion:** These findings suggest that female runners require more effort to keep the same speed as male runners. This study may provide valuable insights into the underlying biomechanical factors of the movement patterns at GRFs during running.

## 1 Introduction

Since the 1970s, running has surged in popularity both as a recreational pursuit and a competitive sport (Van Mechelen, 1992; Van Der Worp et al., 2015; Quan et al., 2021). In recent years, the number of females competing in running events has increased significantly (Hollander et al., 2021). In comparative studies of aerobic exercises, running demonstrates a heightened predisposition to overuse injuries in contrast to activities like walking, swimming, and cycling (Francis et al., 2019). The etiology of running-related injuries (RRIs) is multifaceted, with predominant attributions to anatomical, biomechanical factors and training load (Buist et al., 2010; Vannatta et al., 2020; Xu et al., 2022; Zhou and Ugbolue, 2024). Gender has been posited as a potential risk factor, influencing the overall risk of injury (Buist et al., 2010; Van Der Worp et al., 2015; Hollander et al., 2021). Female runners might exhibit a heightened susceptibility to certain RRIs, including patellofemoral pain and tibial stress fractures, compared to male runners (Wright et al., 2015; Almonroeder and Benson, 2017). However, understanding remains limited concerning sex-specific disparities in the etiology of RRIs.

Gender-related differences in kinematics and kinetics during running have previously been reported. Besson et al. (2022) found that female runners showed larger hip and knee joint motion in the non-sagittal plane than male runners. Almonroeder and Benson (2017) also noticed that hip adduction and internal rotation are greater in females than in males. A study conducted by Sinclair and Selfe (2015) showed that among recreational runners, females demonstrated significantly larger extension and abduction moments in the knee joint, as well as greater patellofemoral contact forces and pressures than males, which may relate to the greater risk of patellofemoral pain in female runners. Most studies of gender differences in running biomechanics have focused on lower limb joint biomechanics. Studies examining differences in ground reaction forces (GRFs) between runners of different genders are limited and inconsistent. Bazuelo-Ruiz et al. (2018) conducted a prospective study and found that females have a significantly greater loading rate and peak propulsive force, and a smaller active peak force than males. Isherwood et al. (2021) also observed that females exhibited a greater loading rate than males. However, the findings of a study conducted by Greenhalgh. (2012) indicate that no significant differences in GRF were observed between males and females. During running, runners experience vertical GRF between 1.5 and 3 times their body weight, which is believed to be a significant risk factor for lower limb injuries (Grabowski and Kram, 2008; Logan et al., 2010). Several studies have investigated the correlation between RRIs and GRF parameters. Loading rate, vertical impact force, peak braking force and impulses have all been demonstrated to be associated with RRIs in previous investigations (Zadpoor and Nikooyan, 2011; Napier et al., 2018; Gao et al., 2023). The connection between GRFs and RRIs has inspired researchers to examine the potential of gait retraining as a preventive measure against injuries (Napier et al., 2018; Dempster et al., 2021).

During running training sessions, speed is commonly adjusted and serves as an indicator of the task’s physical intensity (Floría et al., 2019). As running speed increasing, there is often a rise in stride length, frequency, joint range of motion, joint moment, joint load, and vertical impact force (Grabowski and Kram, 2008; Schache et al., 2011; Orendurff et al., 2018). Runners are believed to experience greater forces on their bodies as they run faster. Specifically, within a speed range of 2–7 m/s, runners achieve a longer stride length by producing increased GRFs (Mercer et al., 2005; Hamner and Delp, 2013; Schache et al., 2014). However, not all biomechanical parameters change with increased running speed. Floría et al. (2019) observed no impact of speed on coordination variability when compared to three different running speeds. Girard et al. (2019) accomplished a study on the impact of varying running speeds, from 10 to 25 km/h, on the extent and variation of asymmetry in essential biomechanical aspects. They concluded that the speed of running does not affect the mechanical asymmetry of the lower limb. A prospective study conducted by Muñoz-Jimenez et al. (2015) revealed no significant differences in foot strike patterns, frequencies or percentages between low-speed and high-speed running. While numerous studies have examined running biomechanics at varying speeds, most have focused on a single gender. It remains to be studied how different speeds affect the running mechanics of male and female runners and the gender differences between them.

In summary, current research lacks sufficient evidence regarding the impact of potential confounding factors such as running speed and gender. There are certain questions to answer, including whether females and males adapt differently to varying speed gradients, the effect of speed on GRFs during overground running, and the possibility of predicting speed changes from GRF parameters. Therefore, our study aimed to investigate the differences in GRFs between male and female runners across a range of speeds (10–16 km/h) and to analyze the correlation between GRF parameters and running speed in both genders. We hypothesize that GRF parameters are related to the running speed, and we also hypothesize that GRF parameters are associated with the gender of runners. Comprehensively clarifying the mechanical factors that influence running performance through GRFs is crucial for understanding human locomotor functions. Insights into the key variables critical for faster running will be beneficial for improving performance.

## 2 Materials and methods

### 2.1 Participants

Thirty male (age: 25.80 ± 3.44 years, height: 1.76 ± 0.05 m, body mass: 75.70 ± 6.14 kg) and eighteen female recreational runners (age: 24.89 ± 2.77 years, height: 1.63 ± 0.04 m, body mass: 54.83 ± 5.15 kg) participated in this study. All runners self-identified as rearfoot strike pattern runners. The exclusion criteria for the study were: 1) any lower limb injury within the past 6 months; 2) any low back or lower limb pain during running; 3) less than 3 years of running experience. The study protocol received approval from the institutional review board of Ningbo University, and all runners provided informed written consent before the testing.

### 2.2 Experimental procedures

After 10 min of laboratory familiarization and a warm-up, all runners performed running tests on a 20 m runway at seven speeds: 10, 11, 12, 13, 14, 15, and 16 km/h. Each participant completed three successful trials at each speed (± 2%) on the runway. The trial was considered successful only if runners struck the force plate with their right foot fully on without targeting, and the speed was within 2% of the prescribed running speed. Runners were required to maintain a steady-state speed until they exited the runway. Running speed was measured by two infrared timing gates placed 3 m apart alongside the runway just before and after the force plate. The order of running speeds was non-random for practical reasons (Schache et al., 2011; Orendurff et al., 2018; Aljohani and Kipp, 2020). Adequate rest was provided between speed increases to prevent fatigue. Additionally, our experimenters had monitored the participants’ fatigue levels throughout the process. If a participant had reported experiencing fatigue during the experiment, they would have been allowed to rest before continuing. All runners wore the same natural running shoes to avoid shoe effects. Ground reaction forces (GRFs) were recorded by an in-ground force plate (AMTI, Watertown, MA, United States) located in the middle of the runway. Data was collected by the force plate using a sampling frequency of 1,000 Hz.

### 2.3 Data analysis

GRFs for each runner were underwent processing using a Butterworth fourth-order low-pass filter with a cut-off frequency set at 20 Hz via MATLAB software (Vision 2019b; The MathWorks, Inc., Natick, MA). Foot strikes were determined by identifying moments when the vertical GRF exceeded a threshold of 20 N, which indicated the beginning of the initial contact phase and the toe-off phase (Jiang et al., 2021). Then, vertical and anterior-posterior GRFs were normalized relative to each runner’s body weight (BW). Due to high variability within and between subjects, medial-lateral GRFs were excluded from this study (Munro et al., 1987). The GRF variables of interest were extracted and computed from the vertical and anterior-posterior directions, including braking impulse, propulsive impulse, peak braking force, peak propulsive force, vertical impulse, vertical average loading rate (VALR), peak vertical impact force and peak vertical impact force. Based on prior investigations focusing on running GRFs, the selection of these variables as the most related components for analysis (Logan et al., 2010; Zadpoor and Nikooyan, 2011; Jiang et al., 2021).

VALR was calculated by determining the average slope between 20% and 80% of the vertical GRF at the first peak (Yu et al., 2021). The first and second vertical GRFs were defined as the peak vertical impact force and active force, respectively. The anterior-posterior GRF represented the braking phase with negative values and the propulsion phase with positive values (Bazuelo-Ruiz et al., 2018; Jiang et al., 2021). For each direction of interest, impulses were computed from the area between the zero line and the GRF curve, which were calculated using the trapezoidal integration approach as follows:

$$Impulse=\sum_{i=1}^{n-1}\frac{1}{2}\left(F_{i+1}+F_{i}\right)\times \left(t_{i+1}-t_{i}\right)$$

In this equation, n is the number of frames, i is the i -th frame, F is the ground reaction force and t is the time value.

### 2.4 Statistical analysis

Average data for each participant were included in the analysis. The normality of the GRF variables was checked via Shapiro-Wilk tests. Pearson’s correlation coefficients were computed to evaluate the relationship between GRF variables and running speed. Correlations were defined as: no relationship or little (r ≤ 0.25), low to fair (0.25 < r < 0.50), moderate to good (0.50 < r < 0.75), and strong (r ≥ 0.75) (Portney and Watkins, 2009). The significance level for determining whether a correlation is statistically significant was set at 0.05. To further determine the level of variance in running speed that was explained by the specific GRF variables, two stepwise linear regressions were performed (one for males and one for females). The discrete GRF variables that were significantly correlated with the running speed were input into one model as the independent variables, while the running speed was considered the dependent variable. The criteria for entering or removing variables from the model were set at alpha levels of 0.05 and 0.10, respectively. The data were analyzed via SPSS software (version 25.0, IBM Corporation, Armonk, NY, United States).

Meanwhile, both vertical and anterior-posterior GRFs were normalized into 101 data points by using the cubic spline interpolation approach to represent stance phase (from 0% to 100%). Given the one-dimensional time-varying characters of GRF curves, a two-tailed independent *t*-test with statistical parametric mapping (SPM) analyses was used to determine gender differences in each running speed, and a one-way repeated measures ANOVA with SPM was used to determine the main effect of running speed in both males and females.

## 3 Results

**FIGURE 1:**
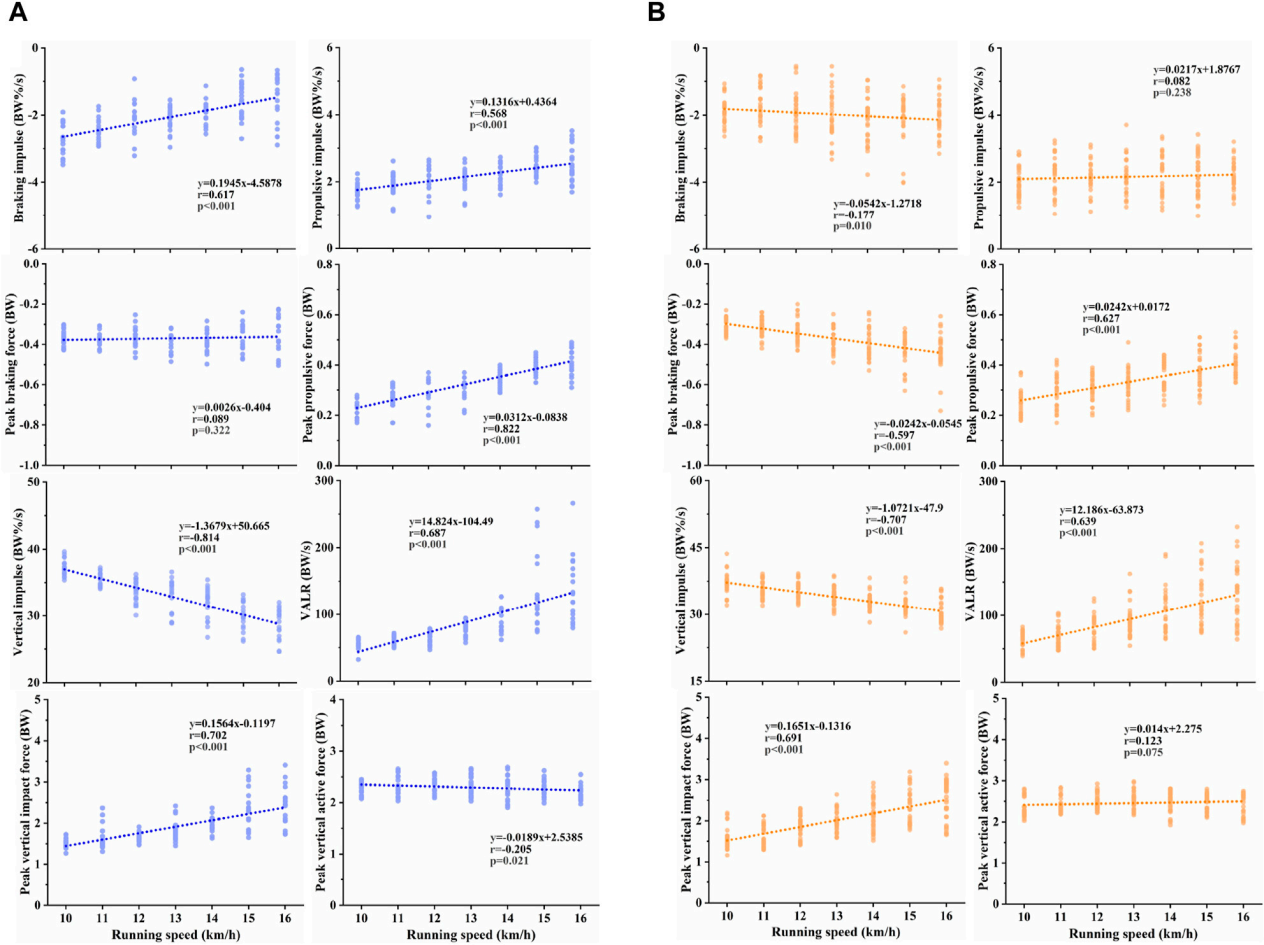
Correlation between running speed and ground reaction force (GRF) variables: braking impulse, propulsive impulse, peak braking force, peak propulsive force, vertical impulse, vertical average loading rate (VALR), peak vertical impact force and active force for **(A)** female runners, and **(B)** male runners.

The relationship between discrete GRF variables and running speed is detailed in Figure 1. In female runners, seven variables showed significant correlations with running speed. Specifically, braking impulse (r = 0.617, *p* < 0.001), propulsive impulse (r = 0.568, *p* < 0.001), peak propulsive force (r = 0.822, *p* < 0.001), VALR (r = 0.687, *p* < 0.001) and peak vertical impact force (r = 0.702, *p* < 0.001) increased linearly with running speed, whereas vertical impulse (r = −0.814, *p* < 0.001) and peak vertical active force (r = −0.205, *p* = 0.021) decreased linearly. In male runners, six variables were significantly correlated with running speed. Peak propulsive force (r = 0.627, *p* < 0.001), VALR (r = 0.639, *p* < 0.001) and peak vertical impact force (r = 0.691, *p* < 0.001) increased linearly with speed, whereas the braking impulse (r = −0.177, *p* = 0.010), peak braking force (r = −0.597, *p* < 0.001) and vertical impulse (r = −0.707, *p* < 0.001) decreased linearly.

The results of stepwise linear regression analysis for females and males were shown in Tables 1, 2, respectively. For females, the analysis identified peak propulsive force, peak vertical impact force, propulsive impulse, VALR and vertical impulse as the best predictors of the running speed (R^2^ = 0.901, *p* < 0.001), explaining 90% of the variation. For males, the best predictors were vertical impulse, peak vertical impact force, peak propulsive force, braking impulse, VALR and peak braking force (R^2^ = 0.855, *p* < 0.001), accounting for 85.5% of the variance in running speed.

**TABLE 1:** Results of stepwise linear regression for running speed in female runners.

Variables	R	*R* ^2^	Adjusted *R* ^2^	F	*p*
Peak propulsive force	0.820	0.673	0.670	255.245	<0.001
Peak propulsive force + peak vertical impact force	0.917	0.842	0.839	326.931	<0.001
Peak propulsive force + peak vertical impact force + propulsive impulse	0.934	0.872	0.869	276.457	<0.001
Peak propulsive force + peak vertical impact force + propulsive impulse + VALR	0.942	0.888	0.884	240.288	<0.001
Peak propulsive force + peak vertical impact force + propulsive impulse + VALR + vertical impulse	0.949	0.901	0.897	217.968	<0.001

**TABLE 2:** Results of stepwise linear regression for running speed in male runners.

Variables	R	*R* ^2^	Adjusted *R* ^2^	F	*p*
Vertical impulse	0.707	0.499	0.497	207.322	<0.001
Vertical impulse + peak vertical impact force	0.830	0.689	0.686	229.037	<0.001
Vertical impulse + peak vertical impact force + peak propulsive force	0.868	0.754	0.750	210.155	<0.001
Vertical impulse + peak vertical impact force + peak propulsive force + braking impulse	0.914	0.835	0.832	258.860	<0.001
Vertical impulse + peak vertical impact force + peak propulsive force + braking impulse + VALR	0.920	0.847	0.843	225.883	<0.001
Vertical impulse + peak vertical impact force + peak propulsive force + braking impulse + VALR + peak braking force	0.925	0.855	0.851	200.043	<0.001

**FIGURE 2:**
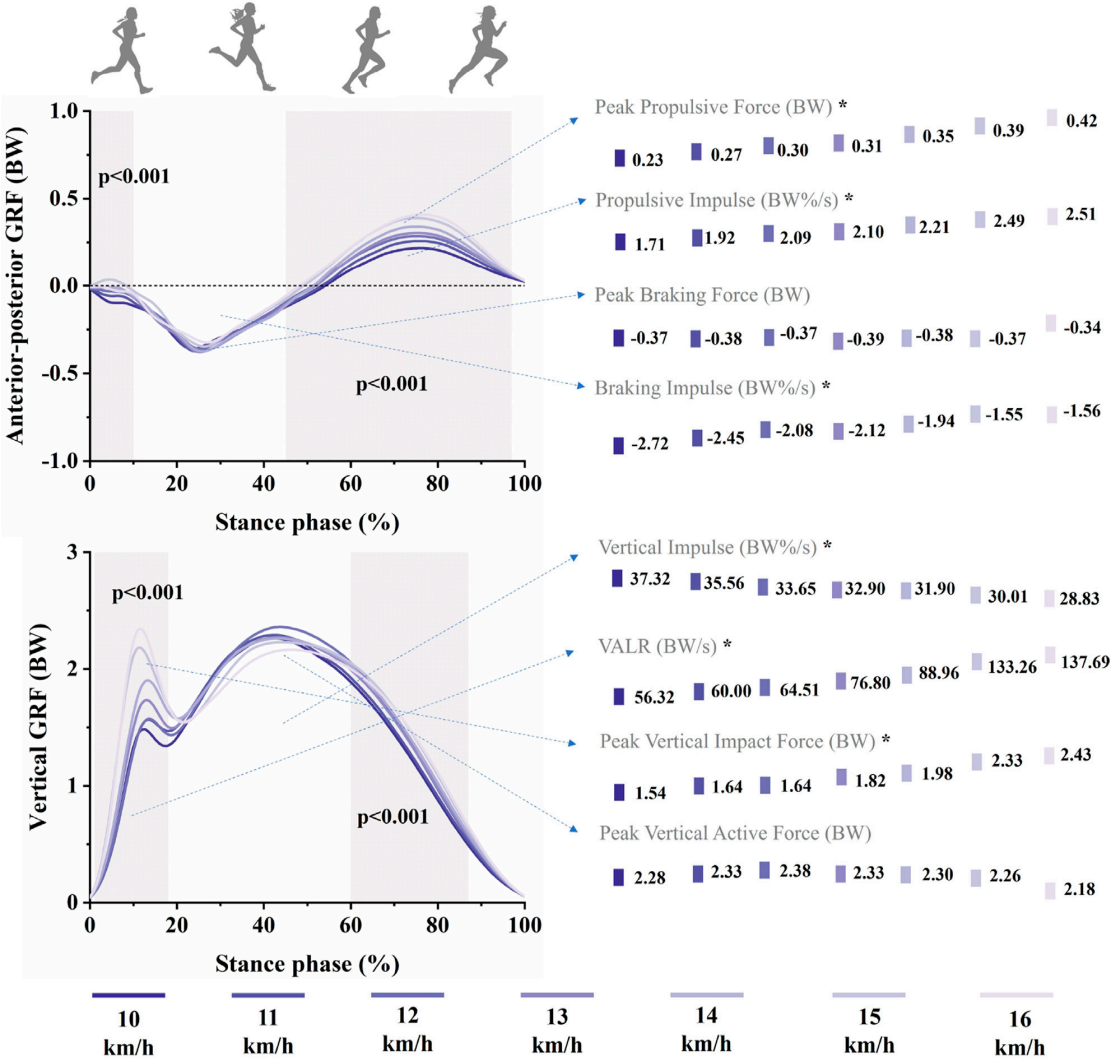
Mean anterior-posterior and vertical GRF waveforms across seven running speeds: 10, 11, 12, 13, 14, 15, 16 km/h for female runners during stance phase. Standard deviations are not presented for further clarity. The grey shaded areas represent significant main effects of running speed from SPM analyses (*p* < 0.05). Point graphs in the figure illustrate mean values of specific GRF parameters at each of the seven speeds. Asterisks indicate significant differences across running speeds (*p* < 0.05).

**FIGURE 3:**
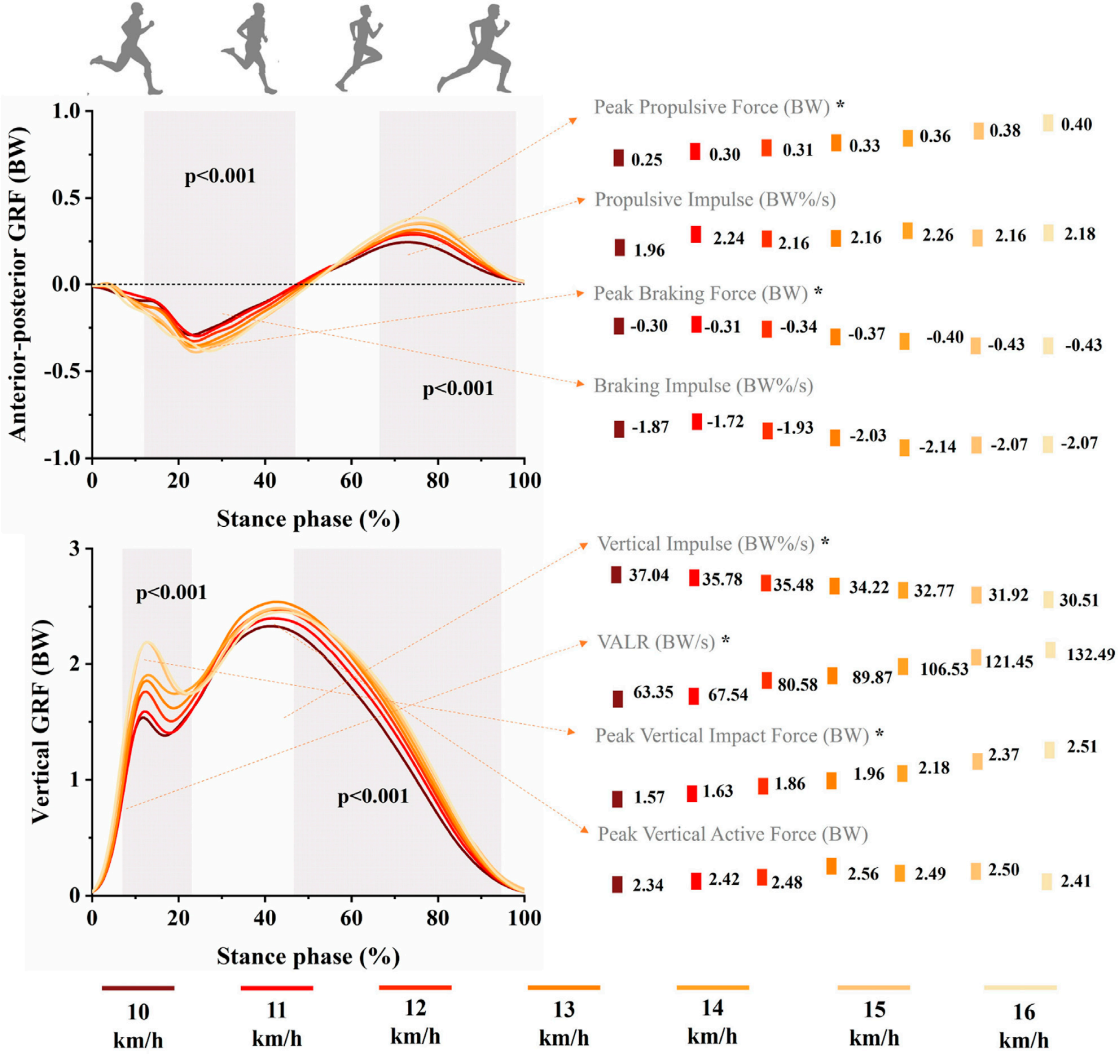
Mean anterior-posterior and vertical GRF waveforms across seven running speeds: 10, 11, 12, 13, 14, 15, 16 km/h for male runners during stance phase. Standard deviations are not presented for further clarity. The grey shaded areas represent significant main effects of running speed from SPM analyses (*p* < 0.05). Point graphs in the figure illustrate mean values of specific GRF parameters at each of the seven speeds. Asterisks indicate significant differences across running speeds (*p* < 0.05).

SPM analyses revealed significant main effects of speed on the anterior-posterior and vertical GRF waveforms for female runners, as shown in Figure 2. Both propulsive force (45%–98%, *p* < 0.001) and vertical force (1%–18%, *p* < 0.001; 60%–88%, *p* < 0.001) increased with running speed. Notably, peak propulsive force, propulsive impulse, braking impulse, vertical impulse, VALR, and peak vertical impact force all demonstrated significant speed main effects. Similarly, for male runners, Figure 3 indicated significant main effects of speed on their anterior-posterior and vertical GRF waveforms. Increased running speed resulted in greater braking force (12%–47%, *p* < 0.001), propulsive force (67%–98%, *p* < 0.001), and vertical force (7%–23%, *p* < 0.001; 47%–95%, *p* < 0.001). Significant main effects of speed were also found in male runners’ peak propulsive force, peak braking force, vertical impulse, VALR, and peak vertical impact force.

**FIGURE 4:**
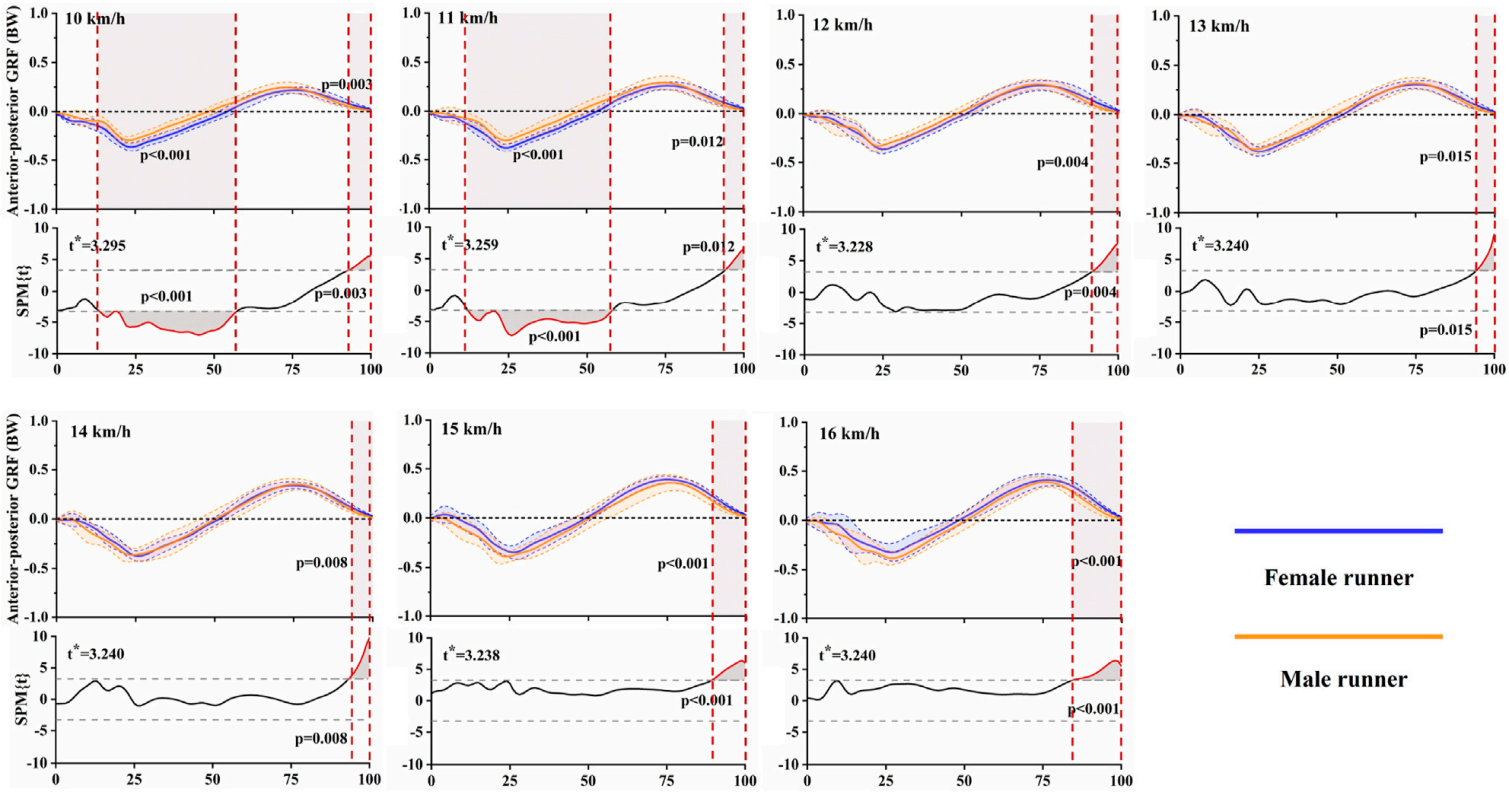
Mean (standard deviation) anterior-posterior GRF waveforms for both female and male runners at each running speed, accompanied by the SPM results. Grey shaded areas represent significant differences between female and male runners during the running stance phase (*p* < 0.05).

**FIGURE 5:**
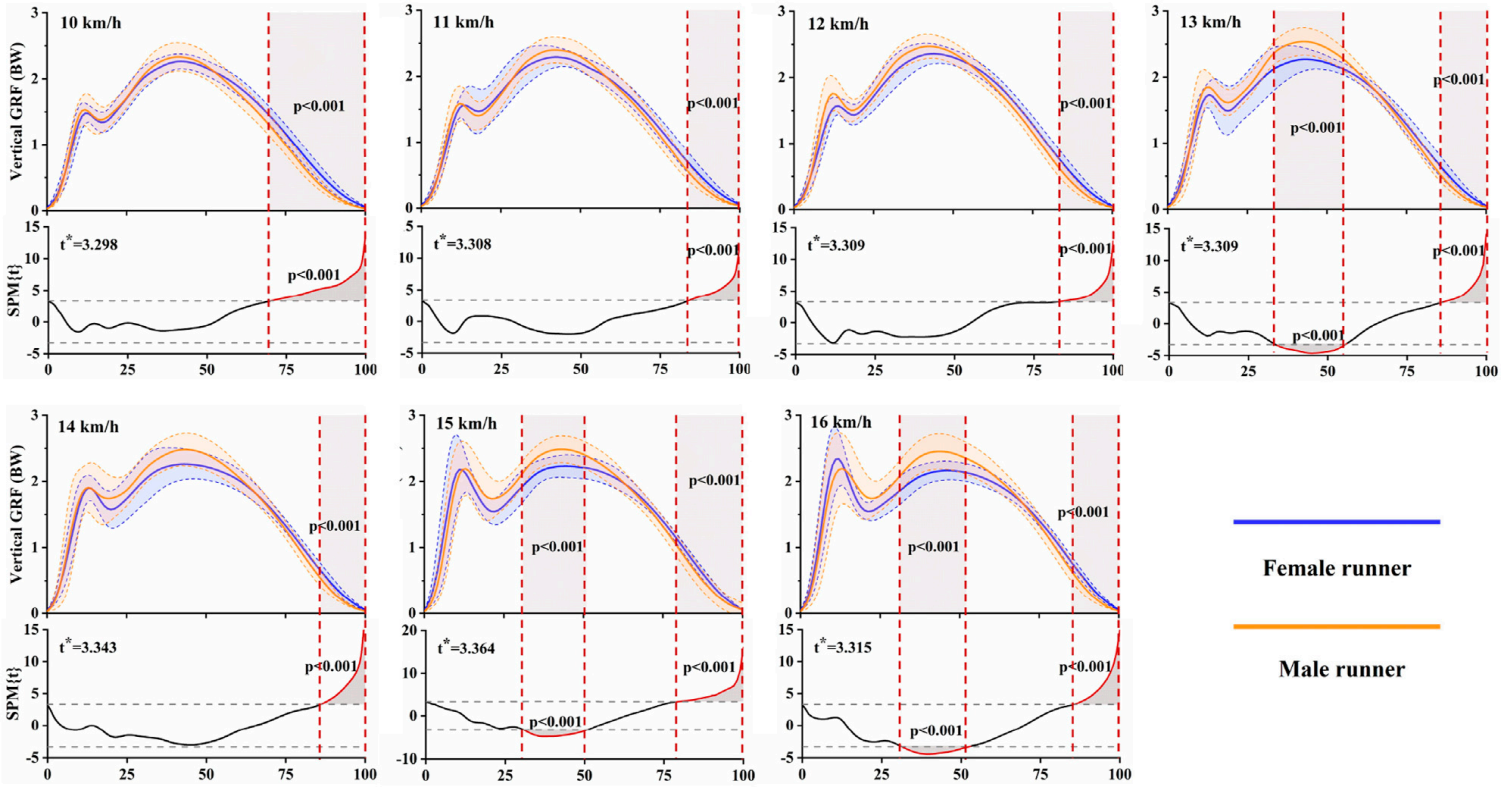
Mean (standard deviation) vertical GRF waveforms for both female and male runners at each running speed, accompanied by the SPM results. Grey shaded areas represent significant differences between female and male runners during the running stance phase (*p* < 0.05).

Figure 4 presented the results of gender differences in anterior-posterior GRFs at each running speed, as determined by independent t-tests and SPM analyses. Females exhibited a larger braking force at speeds of 10 km/h (13%–57%, *p* < 0.001) and 11 km/h (12%–58%, *p* < 0.001). During the later stance phase, females demonstrated more propulsive force than males at all tested speeds. Specifically, this increase was observed during 93%–100% of the stance phase at 10 km/h (*p* = 0.003), 94%–100% at 11 km/h (*p* = 0.012), 92%–100% at 12 km/h (*p* = 0.004), 94%–100% at 13 km/h (*p* = 0.015), 93%–100% at 14 km/h (*p* = 0.008), 89%–100% at 15 km/h (*p* < 0.001), and 84%–100% at 16 km/h (*p* < 0.001). Figure 5 illustrated gender differences in vertical GRFs at each running speed. During the later stance phase, females exhibited higher forces than males at all selected speeds. Specifically, this increase was observed during 70%–100% of the stance phase at 10 km/h (*p* < 0.001), 84%–100% at 11 km/h (*p* < 0.001), 83%–100% at 12 km/h (*p* < 0.001), 86%–100% at 13 km/h (*p* < 0.001), 86%–100% at 14 km/h (*p* < 0.001), 79%–100% at 15 km/h (*p* < 0.001), and 86%–100% at 16 km/h (*p* < 0.001). However, males showed larger force during 34%–56% of the stance phase at 13 km/h (*p* < 0.001), 31%–51% at 15 km/h (*p* < 0.001), and 31%–53% at 16 km/h (*p* < 0.001), which included peak vertical active force.

## 4 Discussion

The primary purpose of our study was to explore gait pattern differences in ground reaction forces (GRFs) between male and female runners across seven running speeds. We hypothesized that specific GRF characteristics would vary between genders across different speeds and that GRFs would correlate with running speed. The primary finding of our study was that female runners exhibit higher propulsive and vertical forces than male runners at all tested speeds. Our findings indicated that distinct running patterns for male and female runners are identifiable through GRFs at each speed, particularly during the later stance phase. We also observed that running speed significantly influences GRFs for all runners, with both genders exhibiting increased trends in early and mid-late stance as speed increased. The stepwise regression analysis revealed that certain discrete GRF variables could predict running speed, thereby providing partial support for our hypothesis.

Distinction in the incidence rates of specific injuries among male and female runners has indicated the necessity to distinguish running mechanics (Van Gent et al., 2007; Boyer et al., 2017; Hollander et al., 2021). The differences in propulsive force between males and females at each running speed suggest that female runners may require more effort to accelerate the body to maintain forward momentum, in order to keep the same speed as male runners. Previous studies also found that propulsive force in females was higher when compared with males at the same speed during running (Bazuelo-Ruiz et al., 2018; Stickley et al., 2018). Females have a larger braking force during the first half of stance at running speeds of 10 km/h and 11 km/h, which indicated that the mass center of females accelerated more backward than males at slower speeds. Faster running speeds require a higher amount of propulsive force, but not necessarily a lower amount of braking force (Morin et al., 2015). Previous studies have analyzed gender differences on parameters extracted from vertical GRF (Greenhalgh, 2012; Bazuelo-Ruiz et al., 2018), but none have prospectively conducted time series curve analysis on GRF throughout the stance phase. A novel finding in this study was that females have higher vertical GRF during the later stance phase at each running speed compared with males. Higher vertical GRF may be considered an inevitable result of needing a higher percentage of available strength to propel the body towards toe-off (Cavanagh and Kram, 1989). Female runners exhibited a greater peak vertical impact force at faster running speeds, which may induce potential shock increases in the musculoskeletal system and thus lead to running-related injuries (Davis et al., 2016; Vannatta et al., 2020). This may provide a potential explanation for the higher patellofemoral pain and tibial stress fracture rates among female runners (Wright et al., 2015; Almonroeder and Benson, 2017).

In 2016, Yokoyama et al. (2016) identified three running speed categories: slow (2.7–2.9 m/s), moderate (3.5–3.7 m/s), and fast (4.4–4.5 m/s) for experienced runners. In this study, we opted for a speed range of 10–16 km/h, corresponding to 2.78–4.44 m/s. This selection spans the spectrum from slow to fast running, facilitating a more comprehensive examination of the effect of running speed on gait mechanisms. Furthermore, the incremental difference of 1 km/h (0.28 m/s) between each chosen running speed allows for a more detailed investigation of the impact of speed on GRFs. As running speed increases, male and female runners exhibit different GRF characteristics. The results of our study demonstrated that running speed had a significant effect on propulsive force during the second half of stance in both females and males. Runners typically exhibit a forward inclination of the trunk, with foot contact striking the ground behind the body’s center of mass. Consequently, from a biomechanical perspective, the aim is to maximize the propulsive component of GRF to maintain faster running speeds (Schache et al., 2014). Additionally, male runners exhibited increased braking force at higher speeds, suggesting greater impact during the braking phase of high-speed running, aligning with previous findings (Hollis et al., 2019). The runner-ground interaction during the braking phase is crucial, playing a significant role in lower extremity injury risk (Davis et al., 2016; Williams et al., 2020; Jiang et al., 2021). At initial ground contact, the lower extremity experiences rapid loading with forces exceeding 1.5 times the runner’s body weight (Grabowski and Kram, 2008; Logan et al., 2010). With increased speed, runners displayed an increased peak vertical impact force, producing greater external loads on their bodies. The forefoot underwent considerable loading. Previous studies investigated running speed as it related to GRFs and found similar correlations (Breine et al., 2019; Hollis et al., 2019). Interestingly, we observed no significant differences in peak vertical active force across speeds. The relationship between peak vertical impact force and lower limb injuries, however, remains a topic of controversy (Napier et al., 2018; Matijevich et al., 2019).

The current results also showed significant correlations (i.e., Pearson correlation and stepwise multiple linear regression) between running speed and the GRF variables, which are compatible with the findings by Breine et al. (2019) and Fukuchi et al. (2017). The GRF variables chosen in this study together explained approximately 90% of the variance associated with increases in running speed. Key contributors and predictors of higher running speeds for both genders included peak propulsive force, vertical impulse, peak vertical impact force and VALR. Schache et al. (2014) suggested that, to achieve higher running speeds, runners tend to exert greater force against the ground rather than increasing the frequency of their strides. This conclusion is also consistent with the higher values we recorded for the propulsive force. Consistent with our hypothesis, variations in vertical GRF were responsive to changes in running speed, indicating the necessity for the legs to generate more vertical force to attain faster speeds. Notably, braking impulse and peak braking force emerged as significant factors only in male runners. This could be explained by the fact that male runners, having relatively larger body weights, experience greater gravity and inertia effects during the braking phase, which emphasizes the importance of the braking phase in their running mechanics (Chang et al., 2000).

This study verified whether running speeds influence the GRF on overground running and whether these likely influences depended on gender differences. However, several limitations must be acknowledged. The selected running speeds are based on absolute values, not relative to each runner’s physiological capabilities. We chose absolute speeds to quantify the impact of speed more accurately on a runner’s GRF and minimize potential biomechanical differences that could arise from differences in relative speeds (Petersen et al., 2014; Jiang et al., 2023). Furthermore, the intervals between the selected running speeds are relatively small. Utilizing speeds based on each runner’s physiological capabilities could have introduced confounding variables into our experimental results. Nevertheless, it is important to acknowledge that this constitutes a limitation of the present study. Notably, even at identical speeds, runners may experience differing physiological intensities (Mo et al., 2020). Another limitation is that the order of running speeds was not randomized. This decision was primarily made to prevent fatigue effects by minimizing the total duration of time spent in the laboratory, which was kept under 2 h (Orendurff et al., 2018). Randomizing the running speeds could be challenging and potentially unsafe, especially when attempting to achieve high running speeds without first gradually progressing through lower speeds (Chang and Cen, 2024; Dorris et al., 2024). It is also important to consider that the observed changes at higher speeds might result from both the external force exerted during ground contact and muscle force production in anticipation of or in response to surface interaction (Logan et al., 2010; Hamner and Delp, 2013; Schache et al., 2014). The knee joint is the most susceptible to injury during running (Vannatta et al., 2020; Zhou and Ugbolue, 2024). While the current study only focuses on GRF, future research will integrate the biomechanics of the knee joint with GRF to investigate the impact of running speed on runners (Adouni et al., 2023; Adouni et al., 2019; AI Khatib et al., 2022). Moreover, our findings are based on data from healthy runners and may not reflect GRF pattern changes in runners with running-related injuries (RRIs). Future research should include runners with RRIs to investigate gender and speed influences on RRI risk factors within this population.

## 5 Conclusion

In conclusion, this study found that increased running speeds (10–16 km/h) on an overground runway led to adaptations in ground reaction forces (GRFs), which were observed in both male and female runners. Despite gender differences, a similar trend in these adaptations was evident. The most significant effects of increased running speed were apparent in the early and late stance phases, with both vertical and anterior-posterior GRFs increasing proportionally. Moreover, female runners exhibited higher propulsive and vertical forces than males in the late stance phase at all speeds, suggesting that females might exert more effort to maintain the same speed as male runners. These findings provide valuable insights into the underlying biomechanical factors of the movement patterns at GRFs during running. Future analyses should focus on enhancing our understanding of the correlation between running-related injuries and gender differences.

## Data Availability

The original contributions presented in the study are included in the article/Supplementary Material, further inquiries can be directed to the corresponding author.

## References

[B1] Adouni M. Alkhatib F. Gouissem A. Faisal T. R. (2023). Knee joint biomechanics and cartilage damage prediction during landing: a hybrid MD-FE-musculoskeletal modeling. PloS one 8, e0287479. 10.1371/journal.pone.0287479 PMC10399834 37535559

[B2] Adouni M. Faisal T. R. Gaith M. Dhaher Y. Y. (2019). A multiscale synthesis: characterizing acute cartilage failure under an aggregate tibiofemoral joint loading. Biomech. Model. Mechan. 18, 1563–1575. 10.1007/s10237-019-01159-9 31069591

[B3] Aljohani M. Kipp K. (2020). Use of self-organizing maps to study sex-and speed-dependent changes in running biomechanics. Hum. Mov. Sci. 72, 102649. 10.1016/j.humov.2020.102649 32721369

[B4] Al Khatib F. Gouissem A. Mbarki R. Adouni M. (2022). Biomechanical characteristics of the knee joint during gait in obese versus normal subjects. Int. J. Environ. Res. Public Health. 2, 989. 10.3390/ijerph19020989 PMC8775559 35055810

[B5] Almonroeder T. G. Benson L. C. (2017). Sex differences in lower extremity kinematics and patellofemoral kinetics during running. J. Sports. Sci. 35, 1575–1581. 10.1080/02640414.2016.1225972 27571504

[B6] Bazuelo-Ruiz B. Durá-Gil J. V. Palomares N. Medina E. Llana-Belloch S. (2018). Effect of fatigue and gender on kinematics and ground reaction forces variables in recreational runners. PeerJ 6, e4489. 10.7717/peerj.4489 29576960 PMC5865467

[B7] Besson T. Macchi R. Rossi J. Morio C. Y. Kunimasa Y. Nicol C. (2022). Sex differences in endurance running. Sports Med. 52, 1235–1257. 10.1007/s40279-022-01651-w 35122632

[B8] Boyer K. A. Freedman Silvernail J. Hamill J. (2017). Age and sex influences on running mechanics and coordination variability. J. Sports. Sci. 35, 2225–2231. 10.1080/02640414.2016.1265139 27937807

[B9] Breine B. Malcolm P. Galle S. Fiers P. Frederick E. C. De Clercq D. (2019). Running speed-induced changes in foot contact pattern influence impact loading rate. Eur. J. Sport Sci. 19, 774–783. 10.1080/17461391.2018.1541256 30394188 PMC9524621

[B10] Buist I. Bredeweg S. W. Bessem B. Van Mechelen W. Lemmink K. A. Diercks R. L. (2010). Incidence and risk factors of running-related injuries during preparation for a 4-mile recreational running event. Br. J. Sports. Med. 44, 598–604. 10.1136/bjsm.2007.044677 18487252

[B11] Cavanagh P. R. Kram R. (1989). Stride length in distance running: velocity, body dimensions, and added mass effects. Med. Sci. Sports. Exerc. 21, 467–479. 10.1249/00005768-198908000-00020 2674599

[B12] Chang H. B. Cen X. Z. (2024). Can running technique modification benefit patellofemoral pain improvement in runners? A systematic review and meta-analysis. Int. J. Biomed. Eng. Technol. 45, 83–101. 10.1504/IJBET.2024.138706

[B13] Chang Y.-H. Huang H.-W. C. Hamerski C. M. Kram R. (2000). The independent effects of gravity and inertia on running mechanics. J. Exp. Biol. 203, 229–238. 10.1242/jeb.203.2.229 10607533

[B14] Davis I. S. Bowser B. J. Mullineaux D. R. (2016). Greater vertical impact loading in female runners with medically diagnosed injuries: a prospective investigation. Br. J. Sports. Med. 50, 887–892. 10.1136/bjsports-2015-094579 26644428

[B15] Dempster J. Dutheil F. Ugbolue U. C. (2021). The prevalence of lower extremity injuries in running and associated risk factors: a systematic review. Phys. Act. Health 5, 133–145. 10.5334/paah.109

[B16] Dorris H. Oh J. Jacobson N. (2024). Wearable movement data as a potential digital biomarker for chronic pain: an investigation using deep learning. Phys. Act. Health 8, 83–92. 10.5334/paah.329

[B17] Floría P. Sánchez-Sixto A. Harrison A. J. Ferber R. (2019). The effect of running speed on joint coupling coordination and its variability in recreational runners. Hum. Mov. Sci. 66, 449–458. 10.1016/j.humov.2019.05.020 31176256

[B18] Francis P. Whatman C. Sheerin K. Hume P. Johnson M. I. (2019). The proportion of lower limb running injuries by gender, anatomical location and specific pathology: a systematic review. J. Sport. Sci. Med. 18, 21–31. PMC6370968 30787648

[B19] Fukuchi R. K. Fukuchi C. A. Duarte M. (2017). A public dataset of running biomechanics and the effects of running speed on lower extremity kinematics and kinetics. PeerJ 5, e3298. 10.7717/peerj.3298 28503379 PMC5426356

[B20] Gao Z. Zhu Y. Fang Y. Fekete G. Kovács A. Baker J. S. (2023). Automated recognition of asymmetric gait and fatigue gait using ground reaction force data. Front. Physiol. 14, 1159668. 10.3389/fphys.2023.1159668 36960154 PMC10027919

[B21] Girard O. Morin J.-B. Ryu J. Read P. Townsend N. (2019). Running velocity does not influence lower limb mechanical asymmetry. Front. Sports Act. Living 1, 36. 10.3389/fspor.2019.00036 33344959 PMC7739712

[B22] Grabowski A. M. Kram R. (2008). Effects of velocity and weight support on ground reaction forces and metabolic power during running. J. Appl. Biomech. 24, 288–297. 10.1123/jab.24.3.288 18843159

[B23] Greenhalgh A. (2012). Gender differences in the kinetics and kinematics of distance running: implications for footwear design. Int. J. Sports. Sci. Eng. 6, 118–128.

[B24] Hamner S. R. Delp S. L. (2013). Muscle contributions to fore-aft and vertical body mass center accelerations over a range of running speeds. J. Biomech. 46, 780–787. 10.1016/j.jbiomech.2012.11.024 23246045 PMC3979434

[B25] Hollander K. Rahlf A. L. Wilke J. Edler C. Steib S. Junge A. (2021). Sex-specific differences in running injuries: a systematic review with meta-analysis and meta-regression. Sports Med. 51, 1011–1039. 10.1007/s40279-020-01412-7 33433864 PMC8053184

[B26] Hollis C. R. Koldenhoven R. M. Resch J. E. Hertel J. (2019). Running biomechanics as measured by wearable sensors: effects of speed and surface. Sports Biomech. 20, 521–531. 10.1080/14763141.2019.1579366 30843475

[B27] Isherwood J. Wang H. Sterzing T. (2021). Running biomechanics and running shoe perception of Chinese men and women. Footwear Sci. 13, 55–67. 10.1080/19424280.2020.1853827

[B28] Jiang X. Xu D. Fang Y. Bíró I. Baker J. S. Gu Y. (2023). PCA of running biomechanics after 5 km between novice and experienced runners. Bioengineering 10, 876. 10.3390/bioengineering10070876 37508903 PMC10376576

[B29] Jiang X. Zhou H. Quan W. Hu Q. Baker J. S. Gu Y. (2021). Ground reaction force differences between bionic shoes and neutral running shoes in recreational male runners before and after a 5 km run. Int. J. Environ. Res. Public Health. 18, 9787. 10.3390/ijerph18189787 34574713 PMC8469130

[B30] Logan S. Hunter I. Hopkins J. T. Feland J. B. Parcell A. C. (2010). Ground reaction force differences between running shoes, racing flats, and distance spikes in runners. J. Sport. Sci. Med. 9, 147–153. PMC3737977 24149399

[B31] Matijevich E. S. Branscombe L. M. Scott L. R. Zelik K. E. (2019). Ground reaction force metrics are not strongly correlated with tibial bone load when running across speeds and slopes: implications for science, sport and wearable tech. PloS one 14, e0210000. 10.1371/journal.pone.0210000 30653510 PMC6336327

[B32] Mercer J. A. Bezodis N. E. Russell M. Purdy A. Delion D. (2005). Kinetic consequences of constraining running behavior. J. Sport. Sci. Med. 4, 144–152. PMC3880881 24431970

[B33] Mo S. Lau F. O. Lok A. K. Chan Z. Y. Zhang J. H. Shum G. (2020). Bilateral asymmetry of running gait in competitive, recreational and novice runners at different speeds. Hum. Mov. Sci. 71, 102600. 10.1016/j.humov.2020.102600 32174449

[B34] Morin J.-B. Slawinski J. Dorel S. Couturier A. Samozino P. Brughelli M. (2015). Acceleration capability in elite sprinters and ground impulse: push more, brake less? J. Biomech. 48, 3149–3154. 10.1016/j.jbiomech.2015.07.009 26209876

[B35] Muñoz-Jimenez M. Latorre-Román P. Soto-Hermoso V. García-Pinillos F. (2015). Influence of shod/unshod condition and running speed on foot-strike patterns, inversion/eversion, and vertical foot rotation in endurance runners. J. Sports. Sci. 33, 2035–2042. 10.1080/02640414.2015.1026377 25812907

[B36] Munro C. F. Miller D. I. Fuglevand A. J. (1987). Ground reaction forces in running: a reexamination. J. Biomech. 20, 147–155. 10.1016/0021-9290(87)90306-X 3571295

[B37] Napier C. Maclean C. Maurer J. Taunton J. Hunt M. (2018). Kinetic risk factors of running-related injuries in female recreational runners. Scand. J. Med. Sci. Sports 28, 2164–2172. 10.1111/sms.13228 29846979

[B38] Orendurff M. S. Kobayashi T. Tulchin-Francis K. Tullock A. M. H. Villarosa C. Chan C. (2018). A little bit faster: lower extremity joint kinematics and kinetics as recreational runners achieve faster speeds. J. Biomech. 71, 167–175. 10.1016/j.jbiomech.2018.02.010 29472010

[B39] Petersen J. Nielsen R. O. Rasmussen S. Sørensen H. (2014). Comparisons of increases in knee and ankle joint moments following an increase in running speed from 8 to 12 to 16km·h−1. Clin. Biomech. 29, 959–964. 10.1016/j.clinbiomech.2014.09.003 25242200

[B40] Portney L. G. Watkins M. P. (2009). Foundations of clinical research: applications to practice. Upper Saddle River, NJ: Pearson/Prentice Hall.

[B41] Quan W. Ren F. Sun D. Fekete G. He Y. (2021). Do novice runners show greater changes in biomechanical parameters? Appl. Bionics Biomech. 2021, 1–8. 10.1155/2021/8894636 PMC7801088 33488769

[B42] Schache A. G. Blanch P. D. Dorn T. W. Brown N. A. Rosemond D. Pandy M. G. (2011). Effect of running speed on lower limb joint kinetics. Med. Sci. Sport Exer. 43, 1260–1271. 10.1249/MSS.0b013e3182084929 21131859

[B43] Schache A. G. Dorn T. W. Williams G. P. Brown N. A. Pandy M. G. (2014). Lower-limb muscular strategies for increasing running speed. J. Orthop. Sport. Phys. 44, 813–824. 10.2519/jospt.2014.5433 25103134

[B44] Sinclair J. Selfe J. (2015). Sex differences in knee loading in recreational runners. J. Biomech. 48, 2171–2175. 10.1016/j.jbiomech.2015.05.016 26054425

[B45] Stickley C. D. Andrews S. N. Parke E. A. Hetzler R. K. (2018). The effectiveness of scaling procedures for comparing ground reaction forces. J. Biomech. 77, 55–61. 10.1016/j.jbiomech.2018.06.021 30041959

[B46] Van Der Worp M. P. Ten Haaf D. S. Van Cingel R. De Wijer A. Nijhuis-Van Der Sanden M. W. Staal J. B. (2015). Injuries in runners; a systematic review on risk factors and sex differences. PLoS ONE 10, e0114937. 10.1371/journal.pone.0114937 25706955 PMC4338213

[B47] Van Gent R. Siem D. Van Middelkoop M. Van Os A. Bierma-Zeinstra S. Koes B. (2007). Incidence and determinants of lower extremity running injuries in long distance runners: a systematic review. Br. J. Sports. Med. 41, 469–480. 10.1136/bjsm.2006.033548 17473005 PMC2465455

[B48] Van Mechelen W. (1992). Running injuries: a review of the epidemiological literature. Sports Med. 14, 320–335. 10.2165/00007256-199214050-00004 1439399

[B49] Vannatta C. N. Heinert B. L. Kernozek T. W. (2020). Biomechanical risk factors for running-related injury differ by sample population: a systematic review and meta-analysis. Clin. Biomech. 75, 104991. 10.1016/j.clinbiomech.2020.104991 32203864

[B50] Williams L. R. Standifird T. W. Creer A. Fong H. B. Powell D. W. (2020). Ground reaction force profiles during inclined running at iso-efficiency speeds. J. Biomech. 113, 110107. 10.1016/j.jbiomech.2020.110107 33181396

[B51] Wright A. A. Taylor J. B. Ford K. R. Siska L. Smoliga J. M. (2015). Risk factors associated with lower extremity stress fractures in runners: a systematic review with meta-analysis. Br. J. Sports. Med. 49, 1517–1523. 10.1136/bjsports-2015-094828 26582192

[B52] Xu D. Quan W. Zhou H. Sun D. Baker J. S. Gu Y. (2022). Explaining the differences of gait patterns between high and low-mileage runners with machine learning. Sci. Rep. 12, 2981. 10.1038/s41598-022-07054-1 35194121 PMC8863837

[B53] Yokoyama H. Ogawa T. Kawashima N. Shinya M. Nakazawa K. (2016). Distinct sets of locomotor modules control the speed and modes of human locomotion. Sci. Rep. 6, 36275. 10.1038/srep36275 27805015 PMC5090253

[B54] Yu L. Mei Q. Xiang L. Liu W. Mohamad N. I. István B. (2021). Principal component analysis of the running ground reaction forces with different speeds. Front. Bioeng. Biotechnol. 9, 629809. 10.3389/fbioe.2021.629809 33842444 PMC8026898

[B55] Zadpoor A. A. Nikooyan A. A. (2011). The relationship between lower-extremity stress fractures and the ground reaction force: a systematic review. Clin. Biomech. 26, 23–28. 10.1016/j.clinbiomech.2010.08.005 20846765

[B56] Zhou H. Ugbolue U. C. (2024). Biomechanical analysis of lower limbs based on unstable condition sports footwear: a systematic review. Phys. Act. Health 8, 93–104. 10.5334/paah.332

